# A common arrhythmia, not so common at an old age

**DOI:** 10.1007/s12471-013-0511-y

**Published:** 2014-01-11

**Authors:** A. Wilde

**Affiliations:** Academic Medical Centre, Amsterdam, the Netherlands

**Fig. 1:**
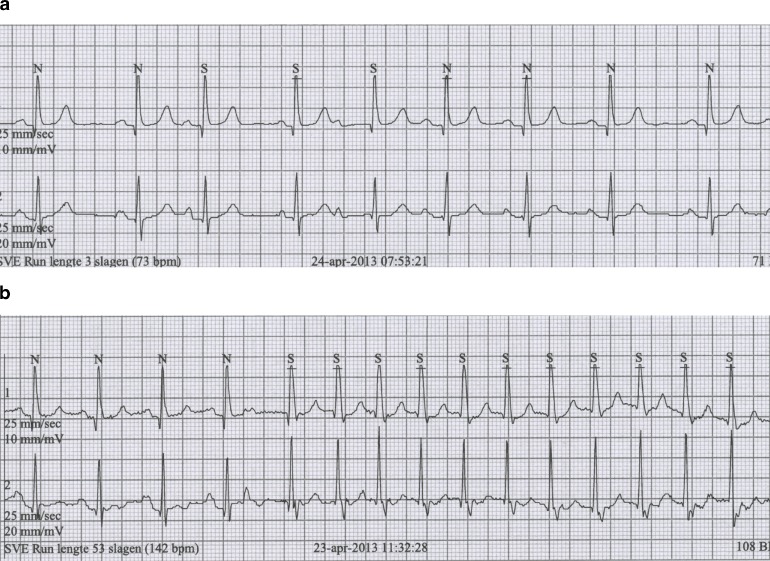
ECG tracings from the Holter monitoring

An 80-year-old lady presents in your outpatient clinic with complaints of palpitations. There is no further cardiac history, her baseline ECG is normal (not shown) and so is her echo. A Holter monitor reveals the following registrations (Fig. 1a and b). What is your diagnosis?

